# Enterovirus D68 Infections Associated with Severe Respiratory Illness in Elderly Patients and Emergence of a Novel Clade in Hong Kong

**DOI:** 10.1038/srep25147

**Published:** 2016-04-28

**Authors:** Susanna K. P. Lau, Cyril C. Y. Yip, Pyrear Su-Hui Zhao, Wang-Ngai Chow, Kelvin K. W. To, Alan K. L. Wu, Kwok-Yung Yuen, Patrick C. Y. Woo

**Affiliations:** 1 Department of Microbiology, The University of Hong Kong, Hong Kong, China; 2 State Key Laboratory of Emerging Infectious Diseases, The University of Hong Kong, Hong Kong, China; 3 Research Centre of Infection and Immunology The University of Hong Kong, Hong Kong, China; 4 Carol Yu Centre for Infection, The University of Hong Kong, Hong Kong, China; 5 Department of Pathology, Pamela Youde Nethersole Eastern Hospital, Hong Kong, China

## Abstract

Despite the recent emergence of enterovirus D68 (EV-D68), its clinical impact on adult population is less well defined. To better define the epidemiology of EV-D68, 6,800 nasopharyngeal aspirates (NPAs) from 2010–2014 were subject to EV-D68 detection by RT-PCR and sequencing of 5′UTR and partial VP1. EV-D68 was detected in 30 (0.44%) NPAs from 22 children and 8 adults/elderlies. Sixteen patients (including five elderly) (53%) had pneumonia and 13 (43%) patients were complicated by small airway disease exacerbation. Phylogenetic analysis of VP1, 2C and 3D regions showed four distinct lineages of EV-D68, clade A1, A2, B1 and B3, with adults/elderlies exclusively infected by clade A2. The potentially new clade, B3, has emerged in 2014, while strains closely related to recently emerged B1 strains in the United States were also detected as early as 2011 in Hong Kong. The four lineages possessed distinct aa sequence patterns in BC and DE loops. Amino acid residues 97 and 140, within BC and DE-surface loops of VP1 respectively, were under potential positive selection. EV-D68 infections in Hong Kong usually peak in spring/summer, though with a delayed autumn/winter peak in 2011. This report suggests that EV-D68 may cause severe respiratory illness in adults/elderlies with underlying co-morbidities.

Since the severe acute respiratory syndrome (SARS) epidemic, intensive research efforts have been made to identify novel causative agents of respiratory tract infections, leading to the discovery of various novel viruses including rhinovirus C (RV-C)^1^,^2^,^3^,^4^,^5^, human metapneumovirus^6^, human bocavirus (HBoV)^7^, and several novel coronaviruses, SARS coronavirus, human coronavirus NL63, human coronavirus HKU1 and Middle East Respiratory Syndrome coronavirus^8^,^9^,^10^,^11^,^12^,^13^,^14^,^15^. On the other hand, existing viruses have continued to emerge or re-emerge to cause respiratory disease epidemics.

Enteroviruses (EVs) are small, non-enveloped, single-stranded, positive-sense RNA viruses that represent one of the 29 genera belonging to the family *Picornaviridae.* The genus now comprises 12 species, EV-A to EV-H, EV-J and rhinovirus A (RV-A) to RV-C (previously named human rhinovirus A to C)^1^,^2^,^3^,^16^,^17^, with >100 immunologically distinct serotypes. In addition, we have also recently described a novel EV species in dromedary camels^18^. EVs belonging to EV-A to EV-D and RV-A to RV-C cause a wide spectrum of diseases in humans^3^,^19^,^20^. EV-A71, one of the most pathogenic EVs, causes large-scale outbreaks of hand-foot-mouth disease (HFMD) with high complication rates in Asia-Pacific region^21^,^22^. Other EVs may also have the potential to cause severe disease and deaths^23^.

EV-D68, a member of the species EV-D, has recently emerged in various countries. In contrast to other EVs which often cause systemic diseases such as HFMD, EV-D68 is mainly associated with respiratory tract infections. The virus was first isolated from throat swab samples of children with bronchiolitis and pneumonia in California in 1962 and referred as “Fermon virus”^24^. Rhinovirus 87 (RV87) and EV-D68 are now considered as the same virus with both RV and EV features (genetically related to other EVs but more temperature- and acid-labile)^25^,^26^. EV-D68 mainly affects infants, children and adolescents, while infections in adults were less commonly reported. Clinical manifestations of EV-D68 infections mainly resemble influenza-like illness, but life-threatening infections were also reported^16^,^27^,^28^,^29^,^30^,^31^. Neurological involvement, ranging from encephalomyelitis to acute flaccid paralysis, was rare, although a recent report highlighted possible association with acute flaccid myelitis^16^,^32^,^33^. Based on sequences of the viral capsid gene VP1, EV-D68 strains are often categorized into 3 distinct genetic groups, clade A to C, in addition to the original Fermon lineage^29^,^33^.

Subsequent to its first isolation, EV-D68 infections have only been rarely reported in the following decades. In US, only 26 cases of EV-D68 infections were detected by passive EV surveillance from 1970 to 2005^34^. However, increasing reports of EV-D68 infections have recently been noted in various countries from Africa, America, Asia and Europe^27^,^28^,^29^,^31^,^35^,^36^,^37^. Since August 2014, more than 1000 cases of EV-D68 have been reported in the United States (US), with at least 14 potentially fatal cases^31^. In Hong Kong, unlike EV-A71 infection which is a notifiable disease, the prevalence of EV-D68 was largely unknown. However, a fatal case of EV-D68 was recently reported in a 10-year-old boy who presented with respiratory symptoms and complicated by encephalitis (http://www.chp.gov.hk/en/view_content/36405.html). To better define the disease impact and clinical spectrum of EV-D68, we examined the clinical and molecular epidemiology of EV-D68 among hospitalized patients with suspected respiratory virus infections in Hong Kong. The partial VP1, 2C and 3D genes of EV-D68 strains were sequenced to study the genetic diversity and evolutionary dynamics.

## Results

### Detection of EV-D68 from NPAs

**Figure 1:**
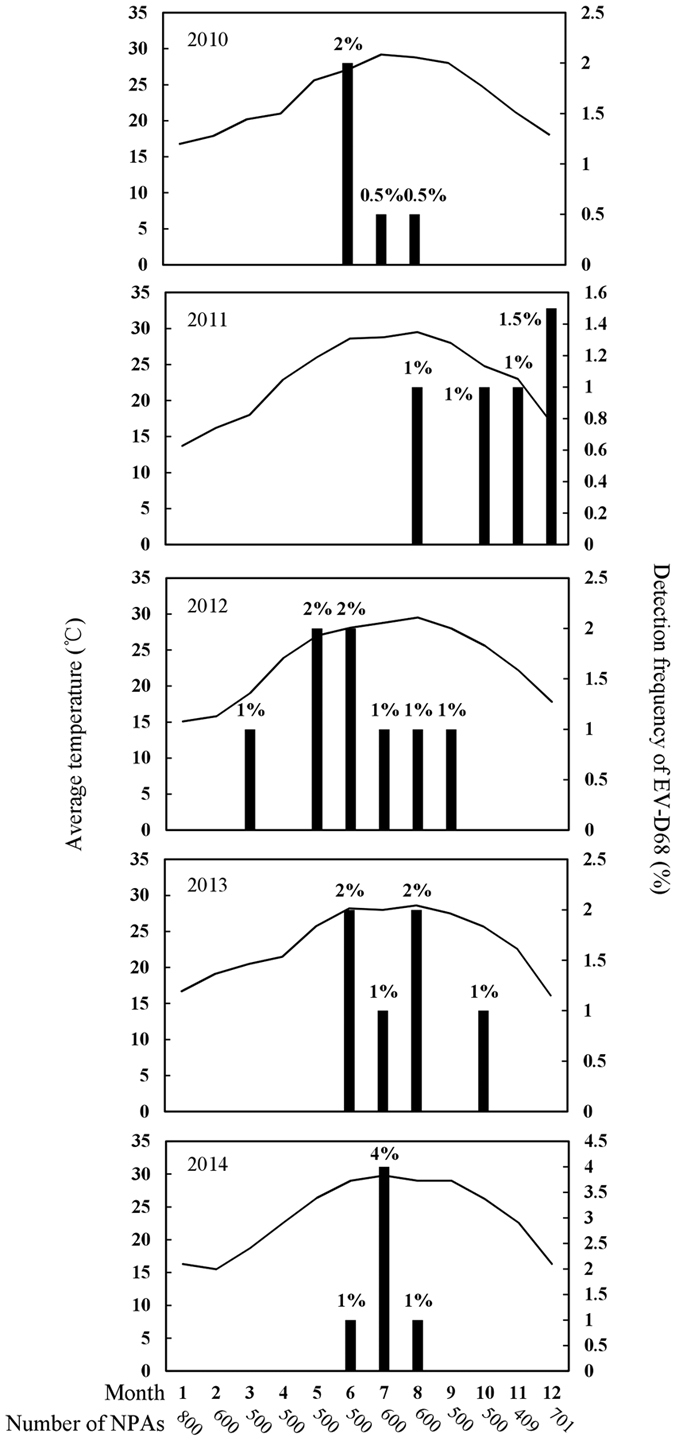
Seasonal distribution of EV-D68 in Hong Kong from 2010 to 2014. Bar graph shows percentage of NPAs positive for EV-D68 in each month and line graph shows monthly average temperatures (°C) in Hong Kong.

A total of 6800 NPAs were subject to EV detection by RT-PCR and sequencing of partial 5′UTR region. Samples positive for EVs were then subject to EV-D68 detection by RT-PCR and sequencing of VP1 region using EV-D68-specific primers. VP1 gene analysis showed that 30 (0.44%) NPAs were positive for EV-D68. The annual incidence rates were 0.27% (4/1500), 0.4% (6/1500), 0.62% (8/1300), 0.46% (6/1300) and 0.5% (6/1200) in year 2010, 2011, 2012, 2013 and 2014 respectively (Fig. 1). The peak season of EV-D68 usually occurred in late-spring/summer (May to August), except in 2011 when more cases were detected in late-autumn/early-winter (October to December).

### Clinical characteristics of patients with EV-D68 infections

A bimodal age distribution was observed among the 30 patients (male: female = 19:11) with EV-D68 infections (Table 1), which included 22 children (<18 years), one 37-year-old adult and seven elderlies (>60 years). Of the seven elderlies, two resided in elderly homes (patients 19 and 23). A 15-year-old adolescent (patient 6) and a 37-year-old adult (patient 13) were also institutionalized. Close contacts of three patients were noted to have recent respiratory illnesses, including siblings of patients 3 and 4, and domestic helper of patient 28. One elderly (patient 20) had recent travel history to mainland China. No epidemiological linkage was identified among the 30 patients.

Twenty-two patients had underlying diseases, such as respiratory diseases and allergies. Most patients presented with acute respiratory illness. Sixteen patients (53%), including five elderlies, presented with pneumonia which represented the most common diagnosis. One elderly (patient 23) with pneumonia was complicated by congestive heart failure. Various radiological abnormalities were observed, including perihilar haziness, focal haziness, infiltrates and consolidation. Three patients (10%) presented with acute bronchiolitis or bronchitis. Exacerbations of small airway diseases were common, including asthmatic attacks (n = 13), febrile wheeze (n = 2) and exacerbation of chronic obstructive pulmonary disease (n = 2). Five patients (17%) presented with upper respiratory tract infection (URTI), including a 63-year-old elderly (patient 21) who was complicated by acute coronary syndrome and shock. Another 2-year-old girl (patient 2) with acute bronchiolitis was complicated by intestinal obstruction. All patients recovered. Except for a 6-year-old girl (patient 29) with influenza C virus co-infection, no respiratory viruses or bacteria were identified from other patients.

### Molecular epidemiology of EV-D68 circulating in Hong Kong

**Figure 2:**
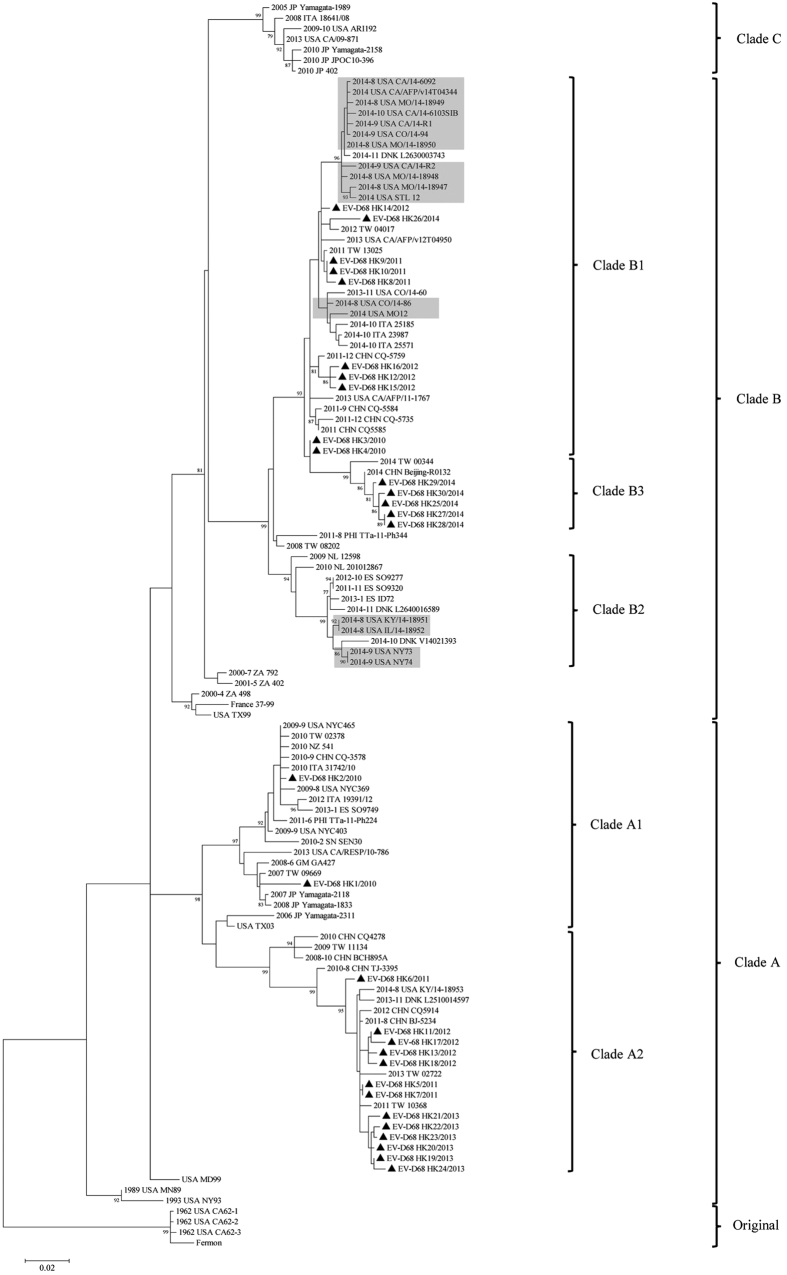
Phylogenetic trees of the partial VP1 regions of EV-D68 strains in Hong Kong. 1004 nucleotide positions were included in the analysis. Strains detected in this study were marked with triangles. Strains detected during the epidemic in US in 2014 were highlighted in light gray. The tree was rooted with the original lineage including the prototype strain Fermon (Genbank accession no. AY426531). The scale bar indicates the estimated number of substitutions per 50 bases.

Phylogenetic analysis of partial VP1, 2C and 3D regions showed that the 30 EV-D68 strains fell into two major clades, clades A and B, corresponding to the two of the three EV-D68 clades described previously (Fig. 2 and Supplementary Fig. 1)^33^,^35^. Previous studies have also revealed further subgroups, such as clade B1 and B2 among strains recently emerged in US^30^,^33^,^38^. Upon VP1 analysis, 15 strains belonged to clade B, some of which being closely related to recent B1 strains from US. Five strains detected in 2014 form a potentially new clade, B3, together with strains from Taiwan and mainland China. The other 15 strains belonged to clade A, which can be further divided into two subclades, A1 (n = 2) and A2 (n = 13). Analysis of 2C and 3D sequences showed similar clustering (Supplementary Fig. 1). The four circulating lineages, clade A1, A2, B1 and B3, shared ≥91.8% aa identities to each other in partial VP1 and displayed distinct amino acid polymorphisms in VP1, 2C and 3D (Supplementary Table 2 and Supplementary Fig. 2).

The four clades appeared to dominate in different years. While both clade A1 and B1 were detected in 2010, clade A1 was not detected in subsequent years. Both clade A2 and B1 were co-circulating in 2011 and 2012. However, clade A2 and B was the only circulating lineage in 2013 and 2014 respectively. Moreover, A2 strains circulating in 2013 were clustered together, as with B3 strains in 2014 (Fig. 2), suggesting epidemics from specific lineages. The B3 strains were only distantly related to recent B1 strains in US, suggesting the recent emergence of this novel subclade. Interestingly, adults/elderlies infected by EV-D68 were exclusively associated with clade A2, including five closely related strains from elderlies in 2013. Closely related B3 strains were also noted in six children in 2014.

### Detection of positive selection in VP1 genes

**Figure 3:**
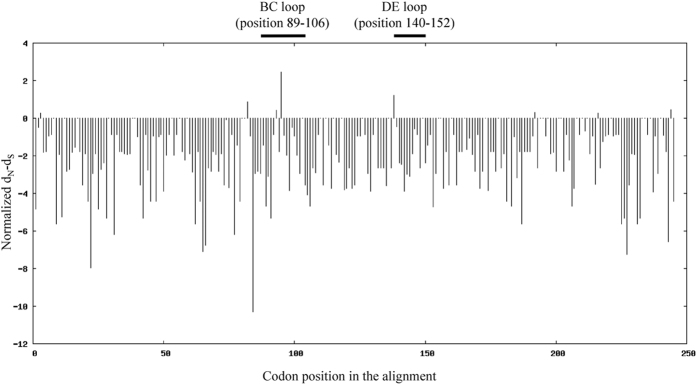
The ratio of nonsynoymous to synonymous substitutions (ω = *d*_*N*_*/d*_*S*_) per codon site in the VP1 region of the 30 EV-D68 strains. Residues with ω > 1 were likely to have evolved under positive selection. Codon position was based on the EV-D68 prototype strain Fermon.

**Figure 4:**
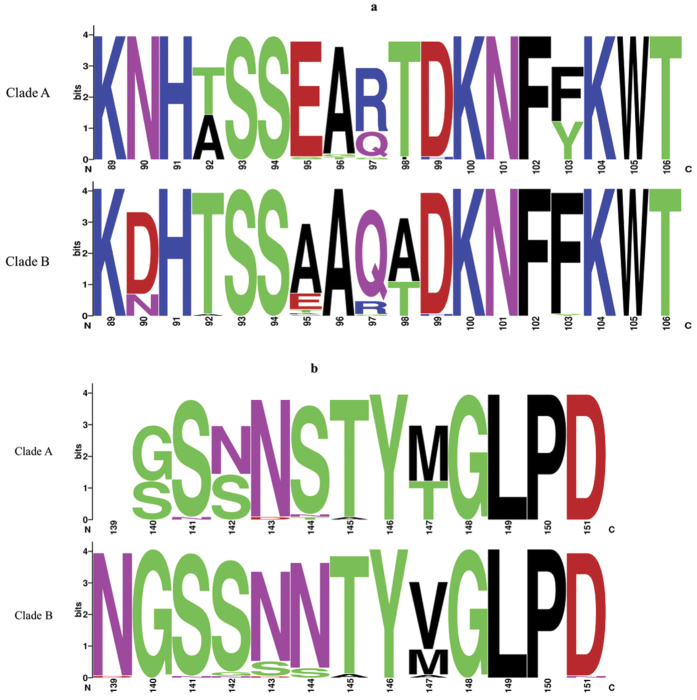
Relative residue abundance in the BC (**a**) and DE (**b**) surface-exposed loops of VP1 among lineage 1 and 2 strains. The graphical representation was generated using WebLogo. The height of symbol indicates the relative frequency of the corresponding amino acid. Amino acid position was based on the EV-D68 prototype strain Fermon.

To assess adaptive evolution of EV-D68, *d*_*N*_/*d*_*S*_ ratios (ω) in VP1 gene across the 30 strains were calculated on codon-by-codon basis. The overall ω was 0.077, with most residues having ω < 1, indicating purifying selection (Fig. 3). Nevertheless, residues 97 and 140 (within BC and DE-surface loops respectively) had ω > 1 but without statistical significance, indicating possible functional constraints during evolution. The dominant aa compositions at BC and DE-surface loops are shown in Fig. 4.

## Discussion

Our study represents the first to demonstrate EV-D68 as a possible cause of severe respiratory illness in adults or elderlies with underlying co-morbidities. In contrast to paediatric population, the role of EV-D68 in adult infections was less clear with only scarce reports. In the Netherlands, EV-D68-related respiratory illness has been described in adults but without clinical details^27^,^29^. Two reports from China have identified 13 and two adults respectively with URTIs due to EV-D68^28^,^39^. A recent report from Denmark identified EV-D68 infections in two adults with URTIs and one adult co-infected by RV with fever, cough and breathing difficulties^30^. In this study, only one elderly presented with URTI alone, while another elderly with URTI was complicated by acute coronary syndrome. The other five elderlies presented with pneumonia, with two having respiratory or cardiac complications. Another 37-year-old adult also presented with pneumonia. Our findings suggested that EV-D68 can cause severe infections in adults or elderlies with underlying diseases.

The EV-D68 infections in adults or elderlies may be underdiagnosed. The conserved 5′UTR is a common target for initial RT-PCR detection of EVs, while specific diagnosis of EV-D68 usually relies on specific RT-PCR assays or VP1 sequencing. In particular, a number of EV-D68 specific diagnostic tools based on real-time PCR assays have been developed recently^40^,^41^,^42^. Although commercially available multiplex PCR assays may include EV-D68, they may lack sensitivity and specificity, and may not differentiate EV-D68 from RVs^42^. Moreover, EV-D68 is seldom included in diagnostic panels for respiratory viruses, while most surveillance programs for EVs focus on stool samples. Inclusion of EV-D68 in routine respiratory virus panels may also help better assess its clinical and public health significance.

The present study also represents the first to describe the epidemiology of EV-D68 infections in Hong Kong. The steady annual incidence observed suggested that EV-D68 has been circulating for at least several years. Unlike EV-A71 with seasonal peaks during summer/fall, as many as 50% of EV-D68 infections may fall outside usual EV peak seasons^34^,^43^,^44^. EV-D68 appears to peak during spring/summer in our population, while a delayed autumn/winter peak was observed in 2011. Therefore, factors other than temperature may also play a role in the seasonality of EV-D68 infection. Lower respiratory illness was common among both elderlies and children, with pneumonia being the most common diagnosis. Many patients were also complicated by exacerbation of small airway disease, in line with the disproportionate presence of asthma among cases in US^45^. Interestingly, a 2-year-old girl (patient 1) had hand-foot-mouth disease which has not been previously reported for EV-D68, although the possibility of co-infection by other EVs cannot be excluded. However, given the limited number of positive samples in this study, further epidemiology studies with inclusion of more cases are required to assess the seasonality, epidemiology and disease impact of EV-D68 in our population.

Our results revealed four lineages of EV-D68, A1, A2, B1 and B3, having circulated in our population and the potential emergence of a new subclade B3 in 2014. Moreover, adults/elderlies were infected exclusively by clade A2, suggesting that this lineage may be emerging in our elderly population. The recent emergence of EV-D68 in different countries may be due to viral evolution of strains/lineages with different antigenicity, with major bifurcation of currently circulating EV-D68 strains dated back to around 1945^33^,^35^,^39^. The aa sequence patterns in BC and DE-surface loops, which determine the antigenic epitopes, may correlate with lineage classification^35^. The four lineages in this study also possessed distinct aa sequence patterns in BC and DE loops (Supplementary Fig. 2a). Moreover, most B strains circulating in 2014 formed a new separate subclade, B3, only distantly related to recent B1 sand B2 trains from US. Other potential B3 strains were also detected in Beijing and Taiwan in 2014 (Fig. 2). On the other hand, some of our B strains detected as early as 2011 were closely related to B1 strains from US. Our findings suggested that the recent epidemic in US may have originated from strains circulating in Asia several years ago, while a new lineage, B3, has only recently emerged in Hong Kong and neighbouring regions. More sequence data are needed to understand the genetic changes that may have driven the emergence of new lineages of EV-D68.

Different EVs are known to utilize difference receptors for cellular entry, which may explain their diverse clinical manifestations and tissue tropisms. In contrast to most EVs with enteric tropism, EV-D68 is seldom detected from stool of infected patients. Although EV-D68 possessed some properties of RVs, it does not utilize the receptors of RVs, namely intercellular adhesion molecule 1 (ICAM-1) or low-density lipoprotein receptor (LDL-R)^46^,^47^. Instead, it shares the same receptor, decay accelerating factor (DAF), with EV-D70 and several echovirus serotypes^25^,^48^,^49^. Interestingly, both EV-D68 and EV-D70 belong to the species EV-D, although EV-D70 is mainly associated with acute hemorrhagic conjunctivitis^19^. In addition, EV-D68 may use sialic acids as receptor, with preferential binding α2–6-linked sialic acids (α2–6 SAs) than to α2–3 SAs, which may suggest higher affinity for upper respiratory epithelium^47^,^50^. Since our results suggest that lower respiratory illness can be common in EV-D68 infections, further studies are required to investigate if some strains or lineages may possess higher affinity for the lower airway.

## Methods

### Ethics statement

The ethical approval was given by the Institutional Review Board of the University of Hong Kong/Hospital Authority Hong Kong West Cluster (UW 04-278 T/600), and the study was conducted in compliance with the principles of the Declaration of Helsinki. Consents for the use of the clinical samples were waived because only left-over samples were used.

### Patients and microbiological methods

All NPAs were collected from hospitalized patients in two regional hospitals in Hong Kong during a five-year period (January 2010 to December 2014), and were tested negative for influenza A and B viruses, parainfluenza viruses types 1, 2 and 3, respiratory syncytial virus, adenovirus, human metapneumovirus, human coronaviruses and human bocavirus^2^.

### RT-PCR for detection of EV-D68 from NPAs

RNA extraction and RT-PCR for EVs were performed using previously described protocols with modifications and primers targeted to the 5′ untranslated region (5′UTR) as shown in Supplementary Table 1^2^,^51^. Both strands of PCR products were sequenced twice with an ABI 3130xl DNA Analyzer (Applied Biosystems), using the PCR primers. Nucleotide sequences were compared to those of known EVs with sequences available in GenBank. Positive samples with sequences belonging to EVs were subject to EV-D68 detection by RT-PCR and sequencing of VP1 gene using EV-D68 specific primers. Samples containing EV-D68 upon VP1 gene sequencing were further subject to 2C and 3D gene sequencing.

### RT-PCR and sequencing of VP1, 2C and 3D gene regions

The partial VP1, 2C and 3D gene regions of EV-D68 strains detected from NPAs were amplified and sequenced using primers shown in Supplementary Table 1 and previously described protocols with modifications^2^,^52^. Phylogenetic trees of each region were constructed using maximum likelihood (ML) method in MEGA6, with bootstrap analysis of 1000 replicates.

### Selective pressure analysis

The number of synonymous substitutions per synonymous site, *d*_*S*_, and non-synonymous substitutions per non-synonymous site, *d*_*N*_, in VP1 were calculated using Nei-Gojobori method (Jukes-Cantor) in MEGA5. Sites under positive selection were inferred using single-likelihood ancestor counting (SLAC) and fixed effects likelihood (FEL) methods as implemented in DataMonkey server (http://www.datamonkey.org). The overall ω (d_N_/d_S_) value was calculated according to NJ trees under the TrN93 substitution model. Positive selection for a site was considered to be statistically significant if *P-*value was <0.1. A mixed-effects model of evolution (MEME) was further used to identify positively selected sites under episodic diversifying selection in particular positions among different clades within a phylogenetic tree even when positive selection is not evident across the entire tree. The relative residue abundance within BC and DE-surface exposed loops were depicted using WebLogo.

### Nucleotide sequence accession number

The sequences of EV-D68 strains have been lodged within GenBank under accession no. KT959173-KT959202 (VP1), KT959113-KT959142 (2C), and KT959143-KT959172 (3D).

## Additional Information

**How to cite this article**: Lau, S. K. P. *et al.* Enterovirus D68 Infections Associated with Severe Respiratory Illness in Elderly Patients and Emergence of a Novel Clade in Hong Kong. *Sci. Rep.* **6**, 25147; doi: 10.1038/srep25147 (2016).

## Supplementary Material

Supplementary Information

## Figures and Tables

**Table 1:** Clinical characteristics of the 30 cases of EVD68 infections.

Patient no.	Month/year	Sex	Age	Underlying disease	Diagnosis	Clade	CXR findings
1	Jun/2010	F	2	None	Hand-foot-mouth disease	A1	NA
2	Jun/2010	F	2	Prematurity, short gut syndrome on TPN, global developmental delay	Acute bronchiolitis, intestinal obstruction	A1	NA
3	Jul/2010	M	11m	None	Pneumonia	B1	Bilateral upper zone haziness
4	Aug/2010	F	5	None	Pneumonia, asthma	B1	Diffuse haziness especially at perihilar region and RLZ
5	Aug/2011	M	7	None	Pneumonia, asthma	A2	Perihilar haziness
6	Oct/2010	M	15	Asthma, ezcema	Asthma	A2	NA
7	Nov/2011	M	6	Eczma, febrile convulsion	Asthma	A2	NA
8	Dec/2011	F	6	None	Pneumonia, asthma	B1	Perihilar haziness
9	Dec/2011	F	4	Down syndrome, AML	URTI	B1	NA
10	Dec/2011	M	8	Hyperpigmentation, asthma	Acute bronchiolitis, asthma	B1	NA
11	Mar/2012	F	77	HT, CA lung	URTI	A2	Blunted right costophrenic angle
12	May/2012	M	6	None	Pneumonia, asthma	B1	Perihilar haziness
13	May/2012	M	37	Severe mental retardation	Pneumonia	A2	Bilateral lower zone infiltrates
14	Jun/2012	M	7	Asthma, ezcema	Pneumonia, asthma	B1	Right perihilar haziness
15	Jun/2012	F	10	Asthma, ezcema	Asthma	B1	NA
16	Jul/2012	F	6	Allergic airway	Pneumonia, asthma	B1	Mild bilateral haziness
17	Aug/2012	F	14m	Prematurity, biliary atresia with LLDT	URTI	A2	NA
18	Sep/2012	F	77	COPD, bronchiectasis, CHF	Pneumonia, COPD exacerbation	A2	RLZ haziness
19	Jun/2013	M	75	NPC, pituitary macroadenoma, panhypopituitarism	Pneumonia	A2	RLZ infiltrates
20	Jun/2013	M	82	HT, old PTB	Pneumonia	A2	RLZ consolidation
21	Jul/2013	M	63	HT, tetralogy of Fallot, monomorphic VT on ICD	URTI, ACS, ICD shock	A2	NA
22	Aug/2013	M	83	HT, COPD, lacunar infarct	Pneumonia, COPD exacerbation	A2	RLZ infiltrates
23	Aug/2013	M	94	DM, AF, IHD, CHF, old PTB	Pneumonia, CHF	A2	RLZ haziness
24	Oct/2013	M	3	Prematurity, cerebral palsy, epilepsy, cortical blindness	URTI	A2	NA
25	Jun/2014	M	4	RSV pneumonia	Acute bronchitis	B3	NA
26	Jul/2014	M	5	Asthma, eczema	Asthma	B1	NA
27	Jul/2014	M	11	Asthma	Pneumonia, asthma	B3	Bilateral hilar haziness, mild peribronchial cuffing
28	Jul/2014	M	11m	None	Febrile wheeze	B3	NA
29	Jul/2014	F	6	Reactive airway, eczema	Pneumonia, asthma	B3	Left perihilar haziness
30	Aug/2014	M	3	None	Pneumonia, febrile wheeze	B3	Perihilar haziness

ACS, acute coronary syndrome; AF, atrial fibrillation; AML, acute myeloid leukemia; CA lung, carcinoma of lung; CHF, congestive heart failure; COPD, chronic obstructive pulmonary disease; DM, diabetes mellitis; HT, hypertension; ICD, implantable cardioverter defibrillator; IHD, ischemic heart disease; LDLT, living donor liver transplantation; NA, no abnormalities; NPC, nasopharyngeal carcinoma; PTB, pulmonary tuberculosis; RLZ, right lower zone; RSV, respiratory syncytial virus; TPN, total parenteral nutrition; URTI, upper respiratory tract infection; VT, ventricular tachycardia.
