# Supplementary material for: Enterovirus D68 Infections Associated with Severe Respiratory Illness in Elderly Patients and Emergence of a Novel Clade in Hong Kong
Source: Sci Rep. 2016 Apr 28;6:25147. doi: 10.1038/srep25147 (PMC4848506; doi:10.1038/srep25147)
Supplement: Supplementary Information [file srep25147-s1.pdf]

Enterovirus D68 Infections Associated with Severe Respiratory Illness in Elderly Patients and Emergence of a Novel Clade in Hong Kong

Susanna K. P. Lau[1-4*+], Cyril C. Y. Yip[1+], Pyrear Su-Hui Zhao[1], Wang-Ngai Chow[1], Kelvin K. W. To[1-4], Alan K. L. Wu[5], Kwok-Yung Yuen[1-4], and Patrick C. Y. Woo[1-4*]

Supplementary Table 1. Primers used in this study

Primer number	Primer sequence (5' - 3')	Genomic region (PCR product size)
LPW 5218	CAAGCACTTCTGTBWCCCCGG	5'UTR (400 bp)
LPW 5228	GAAACACGGACACCCAAAGTAGT	
LPW 24938	GGTTCRTAGCAGCAAAAGATGA	VP1 (843bp)
LPW 24940	GTTTAGGYTTCATGTAAACCC	
LPW 24941	CCAGTTCAAGTCCAAATCTCG	2C (574bp)
LPW 24942	GTGGATCTTGCATTRGTTCT	
LPW 25001	GAGTAYATGGAAGAAGCNGT	3D (502bp)
LPW 25002	GGCATCATAACCAGTGTARTC	

Consensus primers for EVs were used for 5'UTR, while specific primers for EV-D68 were used for VP1, 2C and 3D

Supplementary Table 2. Amino acid identities in the VP1 region between the 30 EV-D68 strains belonging to three lineages

Sequences	Fermon		Clade A1		Clade A2		Clade B1		Clade B3	
	MIN (%)	MAX (%)	MIN (%)	MAX (%)	MIN (%)	MAX (%)	MIN (%)	MAX (%)	MIN (%)	MAX (%)
Clade A1	91.4	92.2	97.5	100						
Clade A2	91.4	93.4	93	98.8	95.5	100				
Clade B1	91.4	92.6	92.2	95.1	91.8	94.7	97.5	100		
Clade B3	92.2	92.6	92.6	94.7	92.6	94.2	97.5	100	99.6	100

a

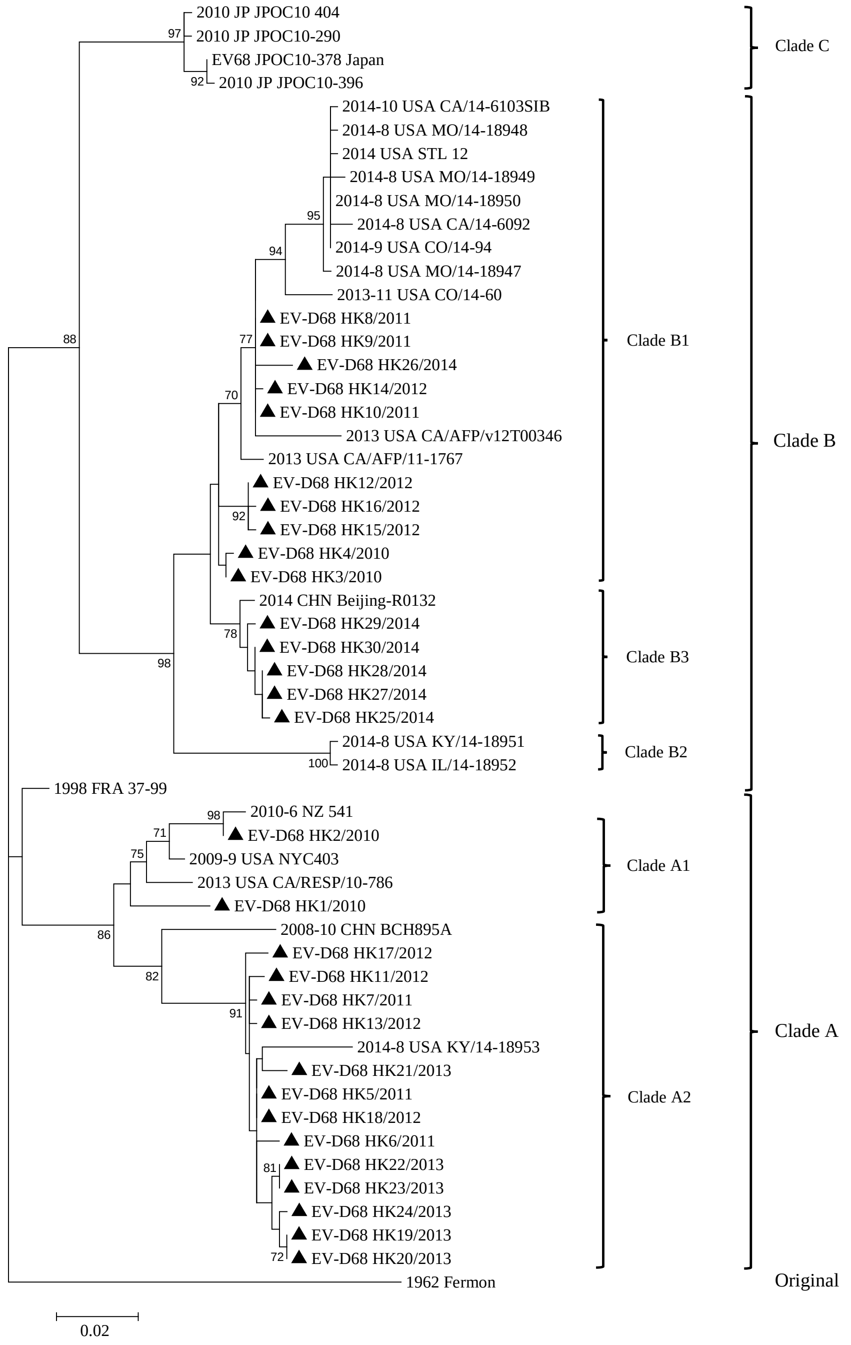

2010 JP JPOC10 404
2010 JP JPOC10-290
EV68 JPOC10-378 Japan
2010 JP JPOC10-396
97
92
Clade C
2014-10 USA CA/14-6103SIB
2014-8 USA MO/14-18948
2014 USA STL 12
2014-8 USA MO/14-18949
2014-8 USA MO/14-18950
2014-8 USA CA/14-6092
2014-9 USA CO/14-94
2014-8 USA MO/14-18947
2013-11 USA CO/14-60
95
94
EV-D68 HK8/2011
EV-D68 HK9/2011
EV-D68 HK26/2014
EV-D68 HK14/2012
EV-D68 HK10/2011
2013 USA CA/AFP/v12T00346
2013 USA CA/AFP/11-1767
77
70
88
EV-D68 HK12/2012
EV-D68 HK16/2012
EV-D68 HK15/2012
92
EV-D68 HK4/2010
EV-D68 HK3/2010
Clade B1
2014 CHN Beijing-R0132
EV-D68 HK29/2014
EV-D68 HK30/2014
EV-D68 HK28/2014
EV-D68 HK27/2014
EV-D68 HK25/2014
78
98
Clade B3
Clade B
2014-8 USA KY/14-18951
2014-8 USA IL/14-18952
100
Clade B2
1998 FRA 37-99
2010-6 NZ 541
EV-D68 HK2/2010
2009-9 USA NYC403
2013 USA CA/RESP/10-786
EV-D68 HK1/2010
98
71
75
Clade A1
2008-10 CHN BCH895A
EV-D68 HK17/2012
EV-D68 HK11/2012
EV-D68 HK7/2011
EV-D68 HK13/2012
2014-8 USA KY/14-18953
EV-D68 HK21/2013
EV-D68 HK5/2011
EV-D68 HK18/2012
EV-D68 HK6/2011
EV-D68 HK22/2013
EV-D68 HK23/2013
EV-D68 HK24/2013
EV-D68 HK19/2013
EV-D68 HK20/2013
86
82
91
81
72
Clade A2
Clade A
1962 Fermon
Original
0.02

b

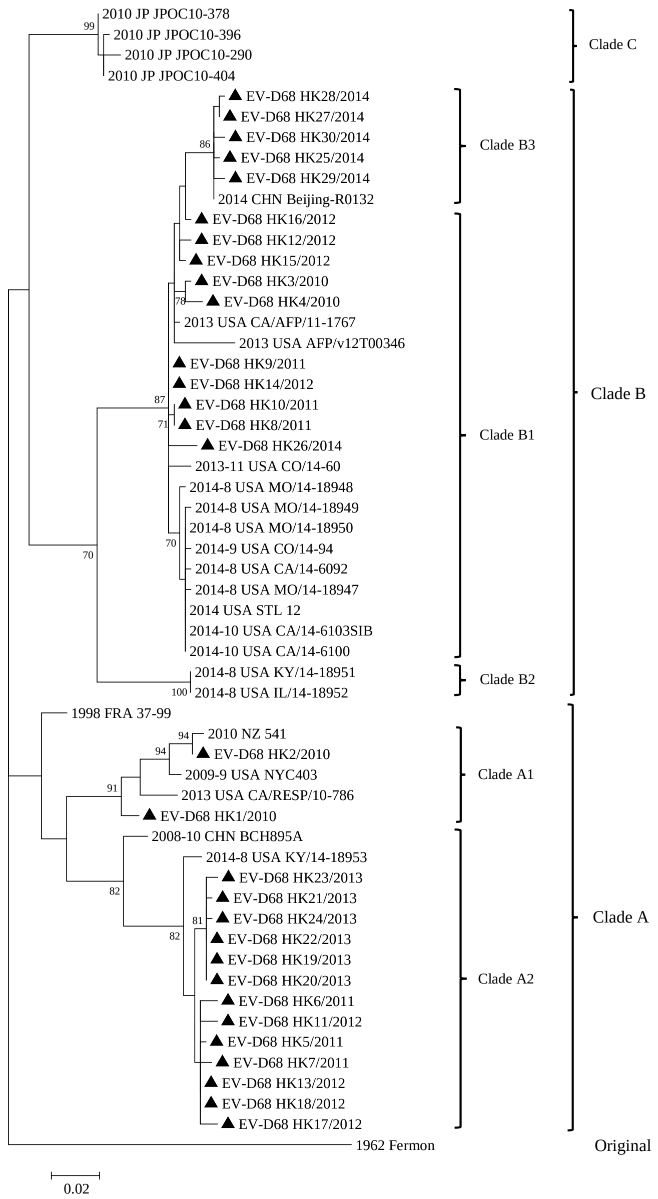

Supplementary figure 1. Phylogenetic trees of the partial 2C (A) and 3D (B) regions of EV-D68 strains in Hong Kong. 549 and 1394 nucleotide positions in 2C and 3D regions were included in the analysis respectively. Strains detected in this study were marked with triangles. The trees were rooted with the prototype strain Fermon. The scale bar indicates the estimated number of substitutions per 50 bases.

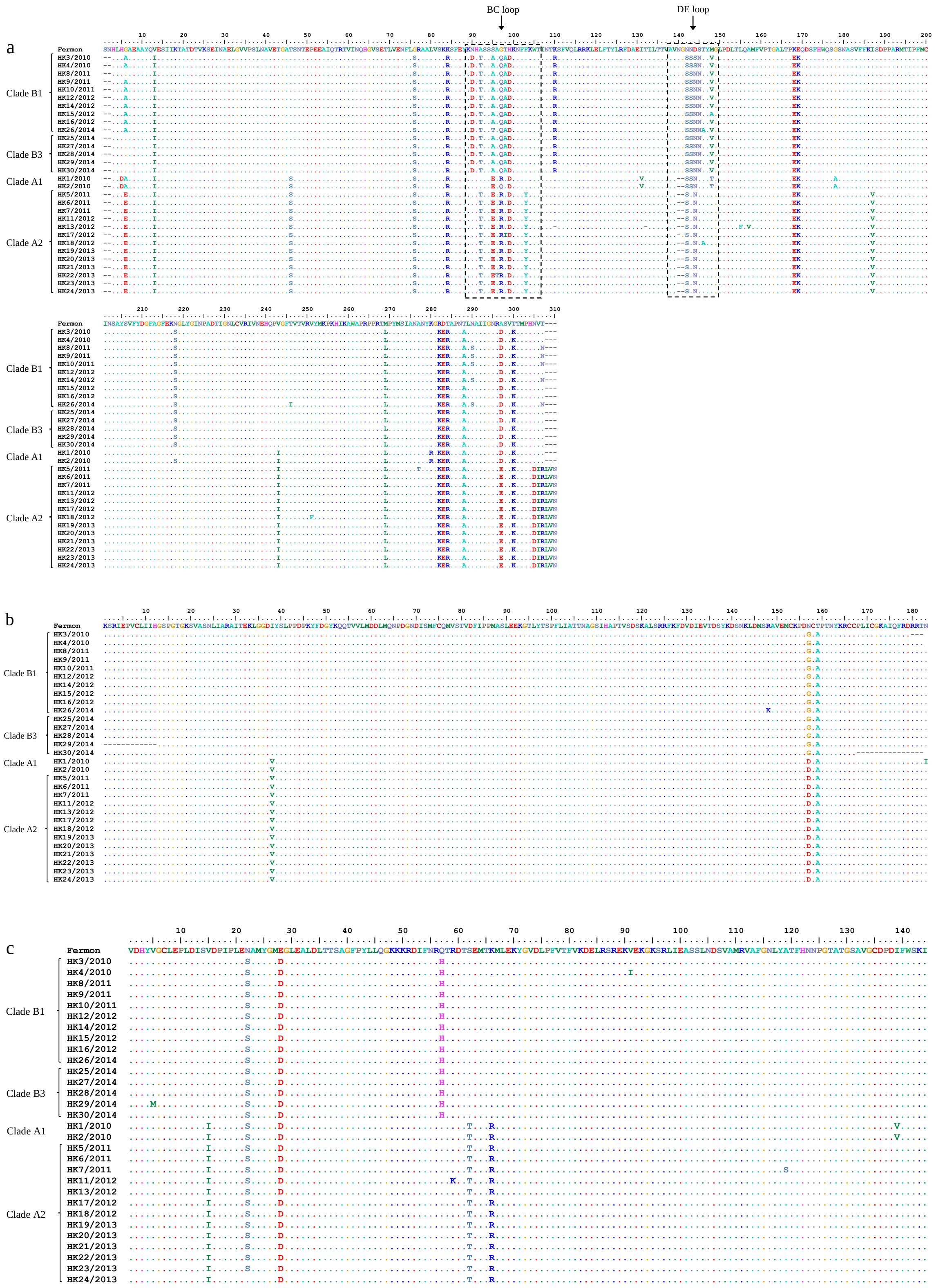

Supplementary figure 2. Multiple sequence alignment of the partial VP1 (A), 2C (B) and 3D (C) regions of the three lineages of EV-D68 strains detected in Hong Kong.

The aa sequences were aligned with the corresponding regions of the prototype strain Fermon. Dots denote the positions where the sequences used for analysis had amino acid residues identical to those of strain Fermon. The BC and DE loops were shown in boxes of VP1 (A).
